# A Digital Model to Simulate Effects of Bone Architecture Variations on Texture at Spatial Resolutions of CT, HR-pQCT, and *μ*CT Scanners

**DOI:** 10.1155/2014/946574

**Published:** 2014-05-18

**Authors:** T. Lowitz, O. Museyko, V. Bousson, W. A. Kalender, J.-D. Laredo, K. Engelke

**Affiliations:** ^1^Institute of Medical Physics, University of Erlangen-Nürnberg, Henkestraße 91, 91052 Erlangen, Germany; ^2^Service de Radiologie Ostéo-Articulaire, Hôpital Lariboisière, Assistance Publique-Hôpitaux de Paris, 2 rue Ambroise Paré, 75010 Paris, France; ^3^Université Paris VII-Denis Diderot, 5 rue Thomas Mann, 75205 Paris, France

## Abstract

The quantification of changes in the trabecular bone structure induced by musculoskeletal diseases like osteoarthritis, osteoporosis, rheumatoid arthritis, and others by means of a texture analysis is a valuable tool which is expected to improve the diagnosis and monitoring of a disease. The reaction of texture parameters on different alterations in the architecture of the fine trabecular network and inherent imaging factors such as spatial resolution or image noise has to be understood in detail to ensure an accurate and reliable determination of the current bone state. Therefore, a digital model for the quantitative analysis of cancellous bone structures was developed. Five parameters were used for texture analysis: entropy, global and local inhomogeneity, local anisotropy, and variogram slope. Various generic structural changes of cancellous bone were simulated for different spatial resolutions. Additionally, the dependence of the texture parameters on tissue mineralization and noise was investigated. The present work explains changes in texture parameter outcomes based on structural changes originating from structure modifications and reveals that a texture analysis could provide useful information for a trabecular bone analysis even at resolutions below the dimensions of single trabeculae.

## 1. Introduction

Quantitative computed tomography (QCT) is an advanced method to measure bone mineral density (BMD) in vivo at various skeletal sites [1]. However, to date the in vivo quantitative analysis of the trabecular bone network remains challenging. For peripheral locations such as the distal radius or tibia dedicated high resolution peripheral QCT (HR-pQCT) equipment with an isotropic spatial resolution of about 130 *μ*m exists [2], but long scan times result in frequent motion artifacts and disturb the analysis of trabecular bone structure [3, 4].

Analysis of the trabecular network imaged with in vivo techniques, predominantly not only with CT but also with MRI or X-ray films, has received a fair amount of attention in the past. In the majority of reported studies, binarization methods were used to separate bone from soft tissue prior to the calculation of histomorphometric parameters [5–7]. However, the spatial resolution of almost all in vivo imaging modalities exceeds the diameter of single trabeculae of about 100 *μ*m to 200 *μ*m [8–10]. Therefore, binarization techniques were avoided in the present work. Instead, texture parameters directly calculated from the gray value distribution of datasets were used.

The texture analysis of trabecular bone is not a new topic and a variety of texture parameters have been used [11–13]. However, a systematic validation of accuracy was rarely included in those studies. Such a validation should include the examination of the dependence of texture on different aspects like structure, BMD modifications, image resolution, and noise. In existing studies usually only the aspect of image resolution was discussed. Textural features were compared among datasets with different spatial resolutions acquired either from different imaging systems like micro-CT (*μ*CT) and HR-pQCT [14], from MR acquisitions with different resolutions [15], or by downsampling of high resolution datasets [16].

In the present work, a digital trabecular bone model consisting of rods and plates is introduced to examine quantitatively the ability to assess the trabecular bone structure at spatial resolutions obtainable with CT, HR-pQCT, and *μ*CT scanners. The model is generic and can be used to simulate typical architectural alterations occurring in osteoporosis or arthritis including the effects of noise and image resolution. As an example in this contribution, we apply the model to quantify entropy, global and local inhomogeneity, local anisotropy, and variogram slope [17]. The aim of the present work is not to simulate architectural changes for a particular disease.

The basic question investigated in the present work is whether variations in bone architecture can be quantified by the use of texture parameters. For this purpose, various structural modifications were applied to the digital bone model. The influence of noise and spatial resolution was included in the investigation. A key topic in the use of texture parameters is their ability to distinguish differences in trabecular architecture from those in BMD. Differences in BMD can easily be measured in vivo using DXA (reproducibility error: 1%-2% [18, 19]) or QCT (reproducibility error: 1%–4% [20–22]) techniques but these techniques cannot differentiate whether BMD changes are caused by changes in tissue mineralization or bone architecture. Thus, two further questions are as follows: Can the use of texture parameters differentiate changes in BMD from changes in BV/TV? And can their use differentiate variations in trabecular architecture that result in the same BV/TV?

The hypothesis of this study is twofold. First, the use of texture parameters is useful to characterize trabecular bone structure using clinical CT equipment, where the spatial resolution is typically inadequate to separate individual trabeculae and, second, based on the dependence of texture parameters on structure at a voxel size of 10 *μ*m, the corresponding characteristics at larger voxel sizes, that is, lower spatial resolution, can be derived.

## 2. Materials and Methods

### 2.1. Basic Trabecular Bone Model

The basic trabecular bone model consists of 1000 × 1000 × 1000 isotropic voxels with an edge length of 10 *μ*m, resulting in a total volume of 1 cm³. The edge length of 10 *μ*m represents the typical voxel size obtainable in *μ*CT datasets. A CT value of 800 HU is assigned to bone voxels and a value of −50 HU to soft tissue voxels. These values are realistically found in trabecular bone. For comparison also CT values of 200 HU were assigned to bone to investigate the texture accuracy at low bone to soft tissue contrasts. The CT value of soft tissue is between CT values of fat (−100) and water (0) [23], as soft tissue surrounding cancellous bone consists mostly of fat and tissue with water equivalent absorption characteristics.

**Figure 1:**
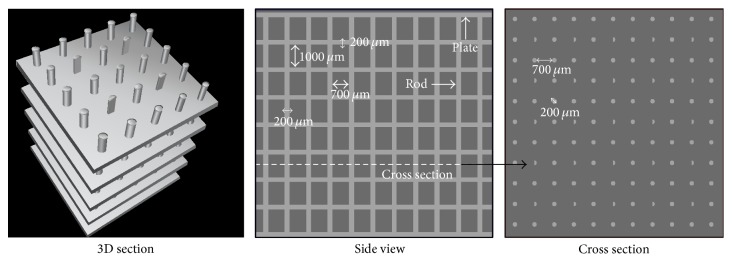
Basic trabecular bone model. For clarity the 3D view only shows a detail of 5 × 5 × 5 rods.

The basic model was built as a combination of rods and plates representing an average human trabecular bone structure [10, 24]. It consists of 11 × 11 cylindrical rods with a diameter of 200 *μ*m and a spacing of 700 *μ*m. The rods are equidistantly interleaved by nine parallel plates with a thickness of 200 *μ*m and a spacing of 1000 *μ*m, which are arranged perpendicular to the rods. Some of the cylinders were cut in half (Figure 1) in order to partially break the symmetry of the structure and to make the model more realistic. This resulted in a bone to tissue volume ratio (BV/TV) of about 20%, which is a typical value found in human epiphyses [25, 26]. Obviously, this model is a simplification of human trabecular bone. However, a more detailed model would not provide generality, limiting its applicability.

Different spatial resolutions (here voxel sizes) were simulated by resampling the structure using a bilinear interpolation implemented in ImageJ [27]. Subsequently, Gaussian noise with a standard deviation of 30 HU, a typical noise level found in in vivo CT acquisitions with medium reconstruction kernels [23], was added to the model. The effect of different reconstruction kernels was simulated by varying spatial resolution and image noise.

### 2.2. Variation of Structure

The structure of the basic model was modified in four fundamental patterns. All modifications were simulated at voxel sizes of 10 *μ*m, 90 *μ*m, and 250 *μ*m, respectively, matching typical voxel sizes of *μ*CT, HR-pQCT, and whole body clinical CT scanners. The four patterns are as follows.

**Figure 2:**
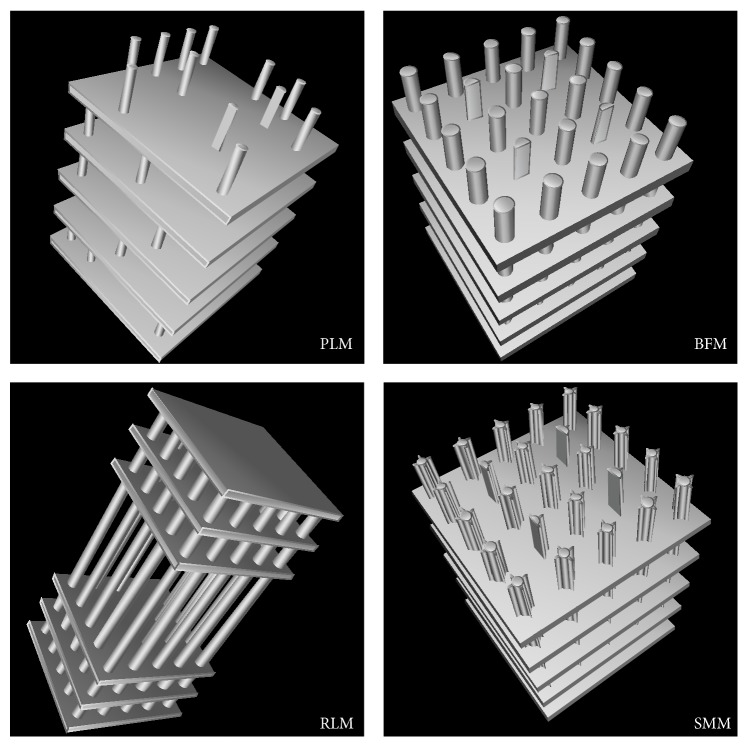
Examples of modifications of the basic model. PLM: 60% of rods are deleted; BFM: rod diameter and plate thickness of 300 *μ*m each; RLM: six plates remaining; SMM: surface irregularities with a width of 80 *μ*m.

(1) A more plate-like structure was simulated. Five models (PLM2–PLM6; PLM: plate-like model) were created in addition to the basic model (PLM1 = basic model) with decreasing numbers of rods (Figure 2 PLM): 20%, 40%, 60%, 80%, and 100% of the rods, respectively, were removed from the models. The trabeculae being removed were chosen randomly (uniform distribution), separately for each layer. Plate thickness was increased with decreasing rod number to keep the overall BV/TV constant. This loss of trabeculae oriented in a specific direction resembles the situation in osteoporosis where in the vertebrae greater bone loss occurs in horizontal compared to vertical trabeculae [28].
(2) An increased bone formation with increased BV/TV was simulated. Five models (BFM2–BFM6, BFM1 = basic model, and BFM = bone formation model) were built with dilated rods and plates (Figure 2 BFM). With every new model, plate thickness and rod diameter were increased by 20 *μ*m, leading to an increase in BV/TV from 20% of the basic model to 32% of the most dilated model (Table 1) with plate thicknesses and rod diameters of 300 *μ*m each. As the global appearance pattern of the bone structure remains quite unchanged, these alterations are expected to have only a little effect on the outcome of the texture parameters, except those which predominantly depend on BV/TV. An increase in BV/TV has been found in osteoarthritis [29].
(3) A more rod-like structure was simulated. Five models (RLM2–RLM6, RLM1 = basic model, and RLM: rod-like model) were created with decreasing numbers of plates and increasing rod thickness (Figure 2 RLM), while BV/TV was kept constant. For the first new model, the central plate was removed and the diameter of all rods was increased. For the models with even fewer plates, the two next central plates were removed. In this way, five additional models with 8, 6, 4, 2, and no plates, respectively, and rod diameters between 200 *μ*m and 500 *μ*m were created. Transformations between plate- and rod-like trabecular structures are present in a variety of musculoskeletal diseases.
(4) Trabecular surface irregularities were simulated. Four cuboids with a length similar to the rod length, a thickness of 30 *μ*m, and a varying width from 20 *μ*m to 120 *μ*m in steps of 20 *μ*m were attached to each rod in 90° steps (Figure 2 SMM: structure modification model). The plates remained unchanged. This resulted in six further models (SMM2−SMM7 and SMM1 = basic model) with increasing surface coarseness and slightly increasing BV/TV (Table 1). The remodeling of trabecular bone is highly load driven (Wolff's law [30]). As changes in loading are a fundamental factor in bone diseases, the detection of superficial trabecular changes may improve the understanding of the bone state during the disease progress.

### 2.3. Variation of Tissue Mineralization

BMD is an important parameter, in particular, as it can be measured in vivo. The underlying causes for BMD changes are a change in trabecular architecture characterized by BV/TV or a change in the mineralization of the trabeculae (tissue mineralization). Both effects can be simulated in our model. It is easy to change the mineralization. In the basic model, CT values for bone were varied between 200 HU and 1200 HU in steps of 200 HU. Generally, tissue mineralization is measured in mg/cm³, but in this work we stick to HU values to be consistent with the values used in the basic model.

### 2.4. Variation of Noise

Pixel noise is a fundamental feature of imaging techniques like CT. Noise increases with increasing absorption by high-attenuating objects, lower mAs settings, and smaller slice thicknesses and highly depends on the reconstruction kernel [23]. As noise strongly affects texture, different noise levels, defined here by the standard deviation of a Gaussian distribution, were applied to the basic model ranging from 5 HU to 50 HU in steps of 5 HU.

### 2.5. Texture Analysis

Five different 3D texture parameters were calculated directly from the gray value distributions of the datasets: entropy, global and local inhomogeneity, local anisotropy, and variogram slope. The inhomogeneity and local anisotropy parameters were already described in [21] but are added here for completeness.

#### 2.5.1. Entropy

Here, the term entropy refers to the Shannon entropy [31], which measures the information content and is given in bits:

(1) $$\begin{array}{c} E=-\sum_{z\in Z}p_{z}\cdot \text{lo}{\text{g}}_{2}p_{z},\;p_{z}=\frac{N_{i}}{N_{\text{VOI}}}, \end{array}$$

where *Z* is the gray value range of the image which is divided into 100 equally distributed partitions (*Z*), called bins, between the minimum and the maximum gray value. With *Z* = 100 the number of empty bins or bins with just a few voxels is probably small. This may not be the case for considerably higher *Z* as the gray value range of imaging datasets is high (12 bits in our case). *p*_*z*_ is the probability of one partition *i*, given as the ratio of the number *N*_*i*_ of voxels in bin *i* and the total number of voxels in the image *N*_VOI_.

#### 2.5.2. Global Inhomogeneity

Global inhomogeneity (Inhom_global_) measures gray value (*g*) fluctuations and is equal to the standard deviation of *g* [21]:

(2) $$\begin{array}{c} {\text{Inhom}}_{\text{global}}=\frac{1}{N_{\text{VOI}}}\overset{N_{\text{VOI}}}{\underset{i=1}{\sum }}\sqrt{{\left(g_{i}-\overset{-}{g}\right)}^{2}}\;, \end{array}$$

where $\overset{-}{g}$ is the mean gray value and *i* iterates over all *n* voxels.

#### 2.5.3. Local Inhomogeneity

In contrast to global inhomogeneity, local inhomogeneity (Inhom_local_) measures local gray value variations which are calculated in a 6-neighborhood [21]:

(3) Inhomlocal=1NVOI ·∑x,y,z  ∈  VOI((162(16gx,y,z(16(gx+1+gx−1+gy+1hhhhhhhhhhhhhhhhhhh+gy−1+gz+1+gz−1)16))2)1/2.

#### 2.5.4. Local Anisotropy

Local anisotropy (*A*) represents the variance of directedness in a local neighborhood [21]. It measures the mean angle difference between the gray value gradient ${\vec{\nu }}_{x,y,z}$,

(4) $$\begin{array}{c} {\vec{\nu }}_{x,y,z}=\left(\begin{array}{c} g_{x+1}-g_{x-1}\\ g_{y+1}-g_{y-1}\\ g_{z+1}-g_{z-1} \end{array}\right), \end{array}$$

of a single voxel and the mean gray value gradient of its 26-neighborhood. Therefore, a local mean gray value gradient ${\overset{-}{\vec{\nu }}}_{\text{local},x,y,z}$ has to be calculated first:

(5) $$\begin{array}{c} {\overset{-}{\vec{\upsilon }}}_{x,y,z}^{\text{local}}=\;\frac{1}{26}\overset{x+1}{\underset{i=x-1}{\sum }}\;\overset{y+1}{\underset{j=y-1}{\sum }}\;\overset{z+1}{\underset{k=z-1}{\sum }}{\vec{\nu }}_{i,j,k},\;i,j,k\;\;\text{distinct}. \end{array}$$

The local anisotropy is then given by

(6) $$\begin{array}{c} A_{\text{local}}=\frac{1}{N_{\text{VOI}}}\overset{N_{\text{VOI}}}{\underset{i=1}{\sum }}\left|∢\left({\vec{\nu }}_{i},{\overset{-}{\vec{\upsilon }}}_{i}^{\text{local}}\right)\right|. \end{array}$$

#### 2.5.5. Variogram Slope

The variogram Var⁡(*d*) describes the mean gray value difference between voxels in a distance *d* to each other [32]:

(7) Var⁡(d)=  12·NNei·∑i=1NVOI∑j=1NNei,i|gi−gj| withNNei=∑i=1NVOINNei,i, ||vi−vj||=d.

For every voxel *v*_*i*_ with gray value *g*_*i*_, the absolute gray value difference to all *N*_Nei,*i*_ neighbor voxels *v*_*j*_ with gray value *g*_*j*_ is calculated. The total sum of absolute gray value differences over all voxels *v*_*i*_ is finally normalized by twice the total number of neighbors as all neighbor pairs are considered twice. The variogram is calculated as a function of increasing voxel distance *d*. The analyses of several CT datasets revealed a plateau of the variogram for *d* > 3. Therefore, here, the slope of Var⁡(*d*) is calculated from linear least squares fit in the interval 1 ≤ *d* ≤ 3 voxels. Obviously, in case of smaller voxel sizes, for example, in *μ*CT data, higher distances can be used. Nevertheless, as the ultimate aim of the present work was the validation of texture parameters measured in clinical CT data *d* was kept constant even when analyzing data with different resolutions. Moreover, the consideration of larger *d* rapidly exceeds acceptable calculation times.

According to their definition and concerning only structural but not mineral changes, it can be expected that entropy and global inhomogeneity mainly depend on BV/TV rather than on the spatial distribution pattern of the trabeculae. Local inhomogeneity, local anisotropy, and variogram slope on the other hand are expected to be rather independent of BV/TV and almost exclusively describe the pattern of the structure.

These five texture parameters remained after a preselection of a higher amount of parameters (e.g., fractal dimension and lacunarity). Excluded parameters showed irregular behavior and high sensitivity on small structure variations.

## 3. Results

### 3.1. Dependence on Structure

**Figure 3:**
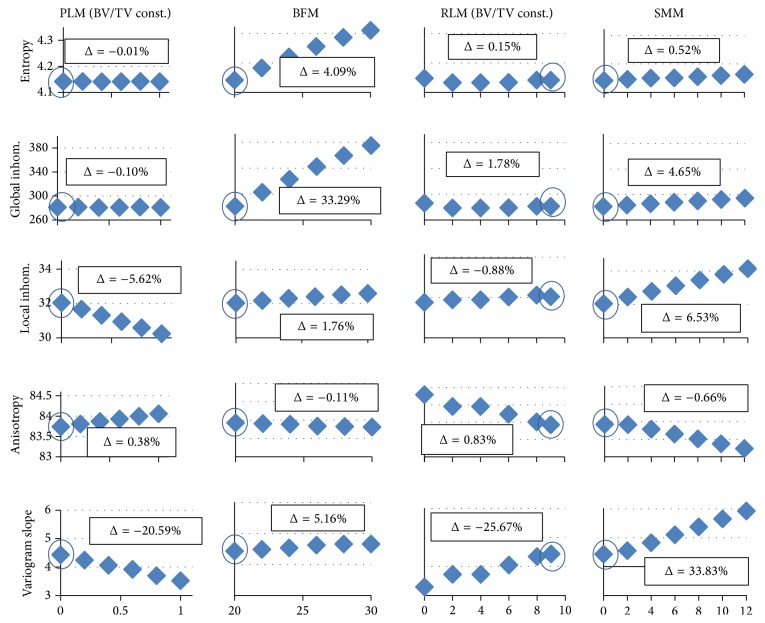
Structure modifications at a voxel size of 10 *μ*m and a noise level of 30 HU. On the *x*-axis, the following measures are plotted. PLM (plate-like model): ratio of rods being deleted in %/100; BFM (bone formation model): BV/TV; RLM (rod-like model): number of plates remaining; SMM (surface modification model): width of surface irregularity in voxels. Results for the basic model are highlighted by circles. % changes between values at the basic model and maximal structure change are given as Δ.

Figure 3 shows the effects of structural modifications on texture parameters at a voxel size of 10 *μ*m and a noise level of 30 HU. Results for the basic model are encircled. Obviously, for a given parameter, the basic model values are identical in all graphs, for example, 4.14 for entropy. For better comparison, for a given texture parameter, the scaling is kept the same for all models. The results look almost identical if bone HU values of 200 instead of 800 are used, although absolute values differ.

For each graph in Figure 3, a linear regression was calculated. An insignificant slope or a nonmonotonic dependency of a texture parameter on structural variations indicates that this parameter may not be suited to pick up structural changes.

The regression slopes of entropy and global inhomogeneity do not significantly differ from zero for PLM and RLM model modifications, in which BV/TV was constant. All other regression slopes from Figure 3 significantly differ from zero. Consequently, and in accordance with their definitions, entropy and global inhomogeneity are independent of structure as long as BV/TV is constant. However, as can be seen from the BFM results, these two texture parameters change with varying BV/TV. There is also a small but significant increase of entropy and global inhomogeneity in the SMM models, in which BV/TV also slightly increases.

In contrast, local inhomogeneity, local anisotropy, and variogram slope depend less on BV/TV as is obvious from the variations for the BFM models. They reflect structural changes for which BV/TV is constant. Slopes are significant for all models but the change is much smaller for the BFM models for which BV/TV changes are the highest. Therefore, there is evidence for the predominant dependence of these three parameters on structure patterns rather than on BV/TV. Consequently, they are able to differentiate differences in trabecular architecture that result in the same BV/TV. Of course, the regression slopes of the four model changes (PLM, BFM, RLM, and SMM) are not directly comparable with each other because their independent variables are different.

Further, a decrease (increase) in local inhomogeneity is always accompanied by an increase (decrease) in local anisotropy and a decrease (increase) in variogram slope. This redundancy suggests the use of only one of these three parameters for the texture analysis of cancellous bone.

### 3.2. Dependence on Tissue Mineralization

**Figure 4:**
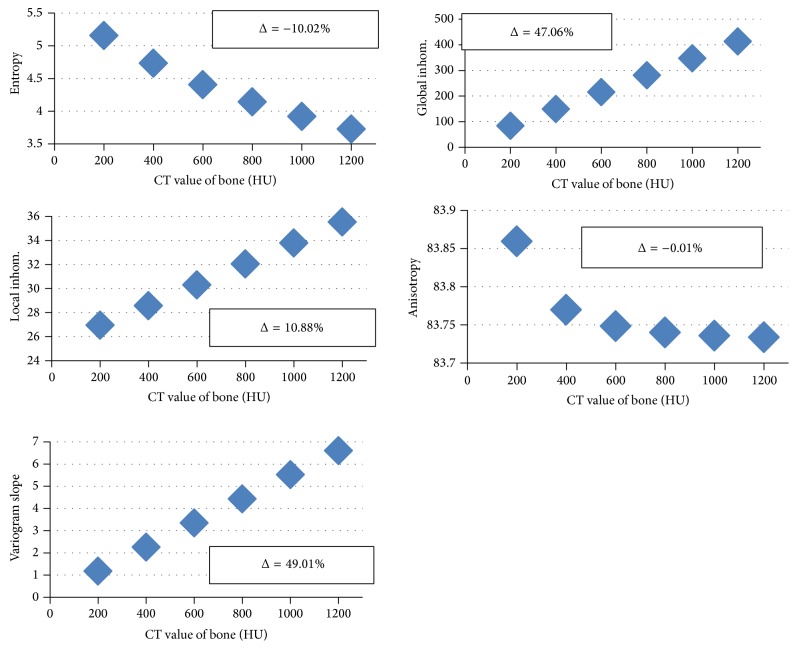
Dependence of texture parameters on tissue mineralization for the basic model at a voxel size of 10 *μ*m and a noise level of 30 HU. Percentage changes between values at 800 HU and 1200 HU are given as Δ.

Texture results for varying tissue mineralization for the basic model at a voxel size of 10 *μ*m are shown in Figure 4. For better comparison, % changes between results for 800 HU and 1200 HU are added to each graph in Figure 4. All parameters show an almost linear dependence on tissue mineralization. Entropy decreases with increasing mineralization and global and local inhomogeneity as well as variogram slope increase. Local anisotropy remains almost constant, whereas global inhomogeneity and variogram slope change very strongly.

### 3.3. Dependence on Spatial Resolution

**Figure 5:**
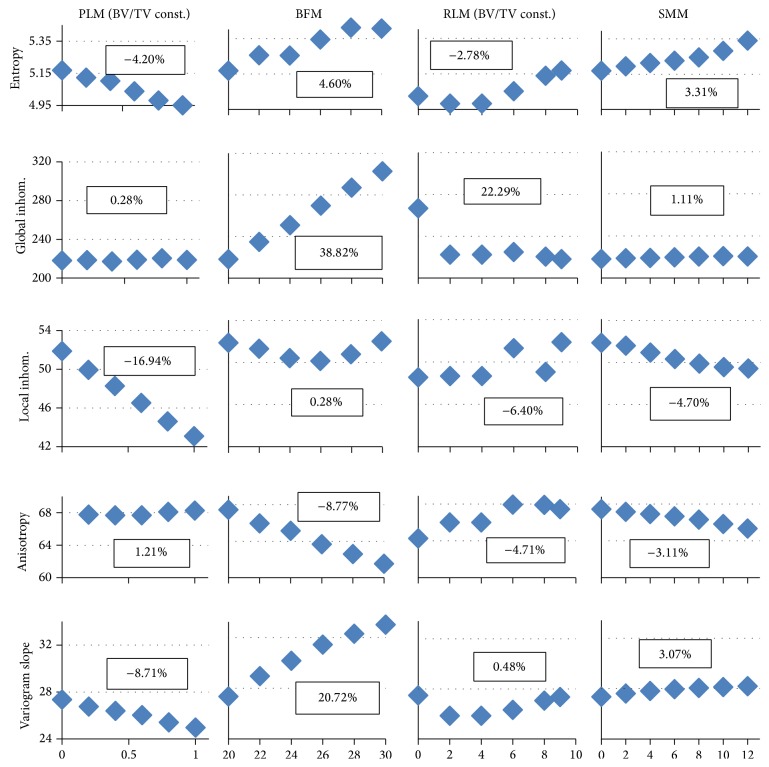
Structure modifications at a voxel size of 90 *μ*m and a noise level of 30 HU. On the *x*-axis, the following measures are plotted. PLM (plate-like model): ratio of rods being deleted; BFM (bone formation model): BV/TV; RLM (rod-like model): number of plates remaining; SMM (surface modification model): width of surface irregularity.

**Figure 6:**
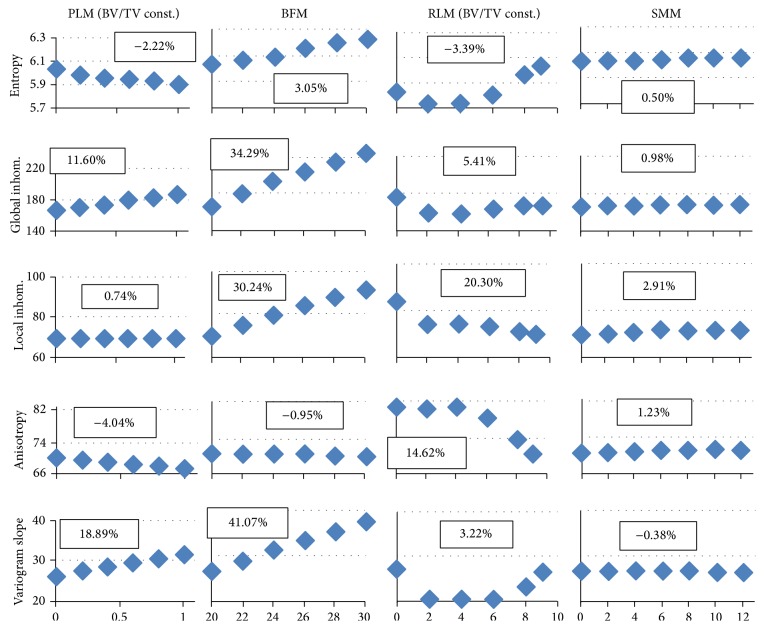
Structure modifications at a voxel size of 250 *μ*m and a noise level of 30 HU. On the *x*-axis, the following measures are plotted. PLM (plate-like model): ratio of rods being deleted; BFM (bone formation model): BV/TV; RLM (rod-like model): number of plates remaining; SMM (surface modification model): width of surface irregularity.

Figures 5 and 6 show the same graphs as Figure 3 but at voxel sizes of 90 *μ*m corresponding to in vivo HR-pQCT and 250 *μ*m corresponding to in vivo QCT datasets. At larger voxel sizes, that is, at lower spatial resolutions, the absolute values of texture parameters change. Values for entropy, local inhomogeneity, and variogram slope increase in comparison to 10 *μ*m for all models whereas values for global inhomogeneity and local anisotropy decrease.

One important question that can be addressed with the digital model is, Is there a limit in spatial resolution for the applicability of texture parameters and, if yes, where? The answer to this question depends on whether (1) the interpretation of texture results shall be independent of spatial resolution or whether (2) an interpretation of texture at a given resolution suffices.

**Figure 7:**
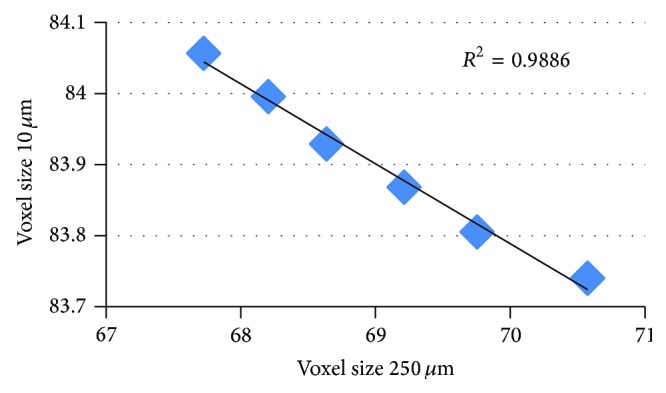
Example of linear regression analysis. Local anisotropy at PLM (plate-like model) changes. Linear correlation between results from voxel sizes (250 *μ*m) and reference voxel size (10 *μ*m).

In order to better understand scenario (1), the results of the texture parameters obtained at voxel sizes of 90 *μ*m and 250 *μ*m were plotted against the results obtained at the reference voxel size of 10 *μ*m. This was performed for all graphs of Figures 5 and 6.  Figure 7 shows one example for anisotropy using the PLM model. If the relationship is not monotonic, the corresponding textural parameter is not suited to detect differences in texture at lower spatial resolutions. Thus, for scenario (1), high *R* ^2^ values obtained from a linear regression applied to the graphs exemplified in Figure 7 are a prerequisite though not being sufficient for an appropriate interpretation of the texture results calculated from datasets with lower spatial resolutions.

*R* ^2^ values are shown in Table 2. The table also lists the ratio of slopes obtained from the linear regressions of a given graph in Figure 5 or Figure 6 and the corresponding graph in Figure 3. This is a figure of merit to investigate whether a decrease in spatial resolution causes a stronger or weaker dependence of texture on structure differences.

A stringent requirement to apply a resolution independent interpretation of texture results would entail that not only the relative change within a given structure modification, that is, for one specific graph in Figure 3, was resolution independent but also the relation among different types of changes, that is, for multiple graphs, was resolution independent. In other words all fields in Table 2 should indicate significance and all slope ratios should be positive. Obviously, this requirement is not fulfilled for any parameter. As a consequence only the resolution dependent interpretation of texture parameters can be used. At different spatial resolutions, different texture parameters or different combinations of texture parameters must be used to characterize or differentiate changes in BV/TV, mineralization, and structure.

### 3.4. Dependence on Noise

**Figure 8:**
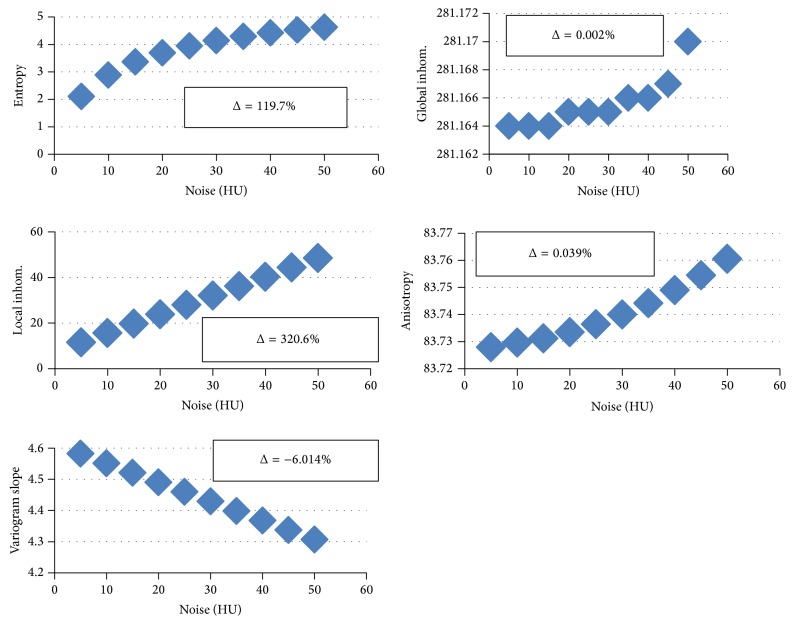
Dependence of texture parameters on image noise for the basic model at a voxel size of 10 *μ*m. Percentage changes between values at noise = 5 HU and noise = 50 HU are given as Δ.

The effect of image noise on texture parameters is shown in Figure 8 for the basic model at a voxel size of 10 *μ*m. The corresponding graphs for the voxel sizes 90 *μ*m and 250 *μ*m are qualitatively similar. Entropy, global and local inhomogeneity, and local anisotropy increase with increasing noise with the noise impact being much higher on local compared to global inhomogeneity. Variogram slope decreases with increasing noise. Local inhomogeneity and variogram slope also linearly depend on noise. Regarding percentage changes, the impact of noise is the lowest on local anisotropy and global inhomogeneity.

### 3.5. Combination of Structural Variations with Changing Noise and Mineralization

In this section several characteristics of the bone models are considered simultaneously. Specifically, the effects of varying structure on texture parameters were quantitatively compared to those caused by noise and by changes in tissue mineralization. As examples, BFM modifications were investigated for entropy and global inhomogeneity and PLM modifications for local inhomogeneity, local anisotropy, and variogram slope.

First, polynomial trend lines (up to 4th order) were fitted to the dependence of texture on noise (Figure 8) and tissue mineralization (Figure 4). All corresponding *R* ^2^ values were high (>0.99). Then entropy and global inhomogeneity were calculated for the basic model, BFM3 and BFM6, and local inhomogeneity, local anisotropy, and variogram slope for basic model, PLM3 and PLM6. For all parameters, absolute differences with respect to the basic model were derived. Finally, those deviations in noise (Δ noise) relative to a noise level of 30 HU and in tissue mineralization (Δ mineralization) relative to 800 HU that would result in the same effect as the structural changes above were calculated. Results are given in Table 3. If numbers are high, then the corresponding texture parameter can pick up structural changes pretty independent of changes in noise and/or mineralization.

## 4. Discussion

The quantitative analysis of the trabecular bone structure increasingly attracts attention in osteoarthritis [33, 34], osteoporosis [12, 35], rheumatoid arthritis [36], and other musculoskeletal diseases. However, it remains unclear what texture parameters really quantify cancellous bone, in particular, if they are applied to lower resolution datasets. Therefore, the aim of the present work was to investigate the ability of texture parameters to quantify trabecular bone changes at different levels of spatial resolution. In contrast to the hypothesis, the results show that the interpretation of texture parameters depends on spatial resolution, because their characteristic response to a change in trabecular structure at 10 *μ*m differs from that at 250 *μ*m. Without a simulation of this response at the spatial resolution under consideration, that is, without a priori knowledge of the expected response, measured texture results cannot be interpreted, as an increase or decrease of the value of a texture parameter can have multiple causes. As a consequence, the use of a realistic digital trabecular bone model is vital for this differentiation task.

The main aim of a texture analysis is to provide information on trabecular structure in addition to BMD, which alone can easily be quantified by DXA and QCT. That implies the question of how strong texture parameters depend on BV/TV, which is the main determinant of BMD as newly formed bone mineralizes up to 70% of its final value within a few days [37, 38]. A strong dependence would limit the additional value of a texture analysis.

At a voxel size of 10 *μ*m, a change of entropy or global inhomogeneity indicates a change in BV/TV but not in structure. As entropy is more noise sensitive global inhomogeneity may be the preferred parameter but it cannot differentiate between changes in BV/TV and mineralization. For this task both parameters must be used in combination as entropy decreases but global inhomogeneity increases with mineralization (see Figure 4). Local anisotropy and variogram slope and to a lesser degree local inhomogeneity are all capable of differentiating two trabecular networks with identical BV/TV but different structure. The variations simulated by the PLM models, more plates and fewer rods, can be better detected than variations simulated by the RLM models, less plates and thinner rods. These are difficult to differentiate from those caused by artificial surface modifications in the SMM models. While a more quantitative analysis is required, probably the measurement of one of these three texture parameters will suffice to allow for the statement that trabecular structure is changed but BV/TV is not. As local inhomogeneity is very sensitive to noise (see Table 3) and its dependence on BFM and RLM variations is somewhat similar, the other two parameters are preferable measurements. If the dependence on mineralization (see Figure 4) is added to the variety of possible modifications, local anisotropy remains the parameter of choice because its variation with mineralization is small compared to those shown in Figure 3. If on the other hand changes in mineralization need not be differentiated from those in BV/TV variogram slope may be a better choice as its percentage variation is much higher than that of local anisotropy.

With increasing voxel size, structure details disappear and the response of texture parameters changes (Figures 5 and 6). The dependence of texture parameters on spatial resolution is affected by the coarseness of the structure. A rather fine structure leads to a more inhomogeneous appearing structure at small voxel sizes, whereas, at large voxel sizes, it leads to a more homogeneous structure due to the resampling process. These homogeneity changes in global appearance strongly affect all texture parameters. As a consequence, with a change in spatial resolution several of the patterns shown in Figure 3 such as variogram slope for the PLM variations or entropy for the RLM variations fundamentally change (compare to corresponding graphs, e.g., in Figure 6) and the question remains, which texture parameters carry structural information at low spatial resolution? In the following only the voxel size of 250 *μ*m will be discussed.

At 250 *μ*m, entropy and global homogeneity still strongly depend on structure independent BV/TV changes; however, in contrast to a voxel size of 10 *μ*m, now entropy also changes with RLM modifications and global inhomogeneity increases with PLM modifications. An increase in entropy or global inhomogeneity is no longer uniquely caused by a BV/TV increase. However, for example, for a concurrent increase of entropy, global and local inhomogeneity, and variogram slope, PLM variations are excluded because this would cause entropy (regarding PLM variations) to decrease. RLM variations are also excluded because local inhomogeneity would decrease. Thus, even at 250 *μ*m, a structure independent change of BV/TV should be identifiable, but a combination of texture parameters has to be measured.

In order to further differentiate changes in tissue mineralization from changes in BV/TV, the information in Figure 6 has to be combined with dependence on noise and mineralization. As can be seen from Figure 4, which looks similar at 250 *μ*m, increases in mineralization increase global and local inhomogeneity and variogram slope; only for entropy the regression slope is negative. Thus, the use of the former three texture parameters would not really allow separating BV/TV and mineralization changes and this may only be possible by carefully analyzing the entropy results in combination. In principle, the data presented in Table 3 are required for this purpose but these are examples only for PLM (local inhomogeneity, local anisotropy, and variogram slope) and BFM (entropy and global inhomogeneity).

Apart from changes in mineralization and BV/TV, changes in texture parameters caused by surface modifications (SMM) are small in relation to other structural changes. At a spatial resolution of 250 *μ*m, these superficial modifications cannot be detected and will not further be considered. Therefore, only two different scenarios remain: a trabecular structure becomes more plate-like (PLM) or more rod-like (RLM). Local anisotropy is well suited to detect transitions to a more rod-like structure (RLM), especially if the transition starts from a hybrid structure (e.g., from RLM 1 in Figure 6, point at the right, to RLM 4) and not from a structure that already contains very few plates (e.g., RLM 4 to RLM 6). Also, local anisotropy is rather stable with respect to changes in noise and tissue mineralization. In order to quantify changes in BV/TV and transitions to a more plate-like structure originating from a hybrid structure (PLM), local inhomogeneity and variogram slope are well suited. Changes in both parameters indicate changes in BV/TV, whereas an exclusive change in variogram slope indicates a transition to a more plate-like structure.

The present study has several limitations. First, a given disease may cause multiple concurrent structural variations. In this case a more complex multivariate analysis will be required. Moreover, real bone structures were not incorporated in the study. Their investigation would complement the simulated data. Also, only five texture parameters were examined. A variety of other parameters exist.

In conclusion, a digital bone model was presented, with which texture parameters for the analysis of cancellous bone in human joints affected by musculoskeletal diseases can be simulated and validated. The presented texture parameters are capable of quantifying changes in the trabecular bone structure even at large voxel sizes of 250 *μ*m achievable in in vivo CT acquisitions. The interpretation of texture parameters strongly depends on spatial resolution. The trends of local inhomogeneity, local anisotropy, and variogram slope vary with different resolution levels. Furthermore, changes in noise and tissue mineralization have to be considered when comparing the texture analysis results among different datasets. Nevertheless, the present work demonstrates that in QCT datasets a texture analysis can complement the BMD analysis to improve the diagnosis and monitoring of patients with musculoskeletal diseases by quantifying changes in the fine trabecular network.

## Figures and Tables

**Table 1:** BV/TV for BFM and SMM structures.

	Model number						
	1	2	3	4	5	6	7
BFM	20.41%	22.44%	24.37%	27.78%	29.62%	32.03%	
SMM	20.41%	20.64%	20.86%	21.08%	21.30%	21.52%	21.74%

**Table 2:** *R* ^2^ values of the linear regression analyses for the voxel sizes 90 *μ*m and 250 *μ*m relative to the reference voxel size (10 *μ*m). Significant linear correlations (*P* < 0.05) are highlighted in bold. In parenthesis: slope ratios obtained from the linear regressions of a given graph in Figure 5 or Figure 6 and the corresponding graph in Figure 3.

Model changes	PLM		BFM		RLM		SMM	
Voxel size/*μ*m	90	250	90	250	90	250	90	250
Entropy	0.00 (−4.4)	0.54 (−7.7)	**0.97 (0.6)**	**0.99 (0.8)**	0.48 (−0.9)	0.57 (−0.5)	**0.98 (0.2)**	**0.97 (0.7)**
Global inhomogeneity	0.48 (0.2)	**0.80 (0.0)**	**1.00 (1.1)**	**1.00 (1.6)**	**0.86 (0.3)**	**0.98 (0.2)**	**0.97 (4.0)**	**0.95 (6.2)**
Local inhomogeneity	**1.00 (−0.1)**	**0.71 (1.0)**	0.16 (0.0)	**1.00 (0.0)**	0.57 ( 0.1)	**0.87 (0.0)**	**0.99 (−0.7)**	**0.96 (0.8)**
Local anisotropy	**0.82 (1.1)**	**0.99 (−0.4)**	**0.98 (0.0)**	**0.90 (0.1)**	**0.92 (−0.2)**	**0.90 (0.1)**	**0.99 (0.3)**	**0.96 (−0.6)**
Variogram slope	**1.00 (−0.4)**	**0.99 (0.2)**	**0.99 (0.0)**	**0.98 (0.0)**	0.18 (0.1)	0.02 (0.0)	**0.95 (1.6)**	**0.94 (−13.1)**

**Table 3:** Deviations in noise and tissue mineralization (both in HU) resulting in identical changes of the texture parameters as the structure variations between the basic model and BFM6 (entropy and global inhomogeneity) or between basic model and PLM6 (local inhomogeneity, local anisotropy, and variogram slope). In parentheses: difference between the basic model and BFM3 or the basic model and BPM3, respectively.

	Entropy	Global inhomogeneity	Local inhomogeneity	Local anisotropy	Variogram slope
Δ noise (10 *μ*m)	4.7 (2.4)	5570.9 (3708.4)	2.2 (0.9)	148.4 (85.3)	149.5 (60.5)
Δ noise (90 *μ*m)	10.4 (5.0)	350.2 (208.8)	16.8 (6.9)	6.2 (1.7)	37.0 (14.9)
Δ noise (250 *μ*m)	12.4 (5.6)	174.2 (109.7)	1.8 (0.4)	32.7 (15.6)	55.1 (20.1)
Δ mineralization (10 *μ*m)	124.1 (59.7)	283.5 (126.0)	209.3 (84.7)	290.7 (138.7)	168.9 (68.3)
Δ mineralization (90 *μ*m)	475.8 (171.3)	333.3 (129.0)	220.3 (90.2)	132.7 (43.3)	69.5 (28.0)
Δ mineralization (250 *μ*m)	218.8 (73.6)	298.0 (132.7)	7.6 (1.6)	334.3 (195.8)	146.3 (53.4)
